# Synthetic regulatory RNAs selectively suppress the progression of bladder cancer

**DOI:** 10.1186/s13046-017-0626-x

**Published:** 2017-10-30

**Authors:** Chengle Zhuang, Xinbo Huang, Changshui Zhuang, Xiaomin Luo, Xiaowei Zhang, Zhiming Cai, Yaoting Gui

**Affiliations:** 1 Guangdong and Shenzhen Key Laboratory of Male Reproductive Medicine and Genetics, Institute of Urology, Peking University Shenzhen Hospital, Shenzhen-Peking University-the Hong Kong University of Science and Technology Medical Center, Shenzhen, 518000 People’s Republic of China; 2 The Department of Biochemistry and Molecular Biology, School of Basic Medical Sciences, Peking University Health Science Center, Beijing, 100191 People’s Republic of China

**Keywords:** Bladder cancer, Human telomerase reverse transcriptase, Artificial miRNA, GAL4

## Abstract

The traditional treatment for cancer is lack of specificity and efficacy. Modular synthetic regulatory RNAs, such as inhibitive RNA (iRNA) and active RNA (aRNA), may overcome these limitations. Here, we synthesize a new iRNA to bind the upstream activating sequence (UAS) of a minimal promoter that drives expression of artificial miRNAs (amiRNAs) targeting MYC, which represses the binding interaction between UAS and GAL4 fusion protein (GAL4-VP64) in GAL4/UAS system. The aRNA driven by a tumor-specific mutant human telomerase reverse transcriptase (*hTERT*) promoter is created to interact with iRNA to expose UAS again in bladder cancer. Without the aRNA, mRNA and protein levels of MYC, cell growth, cell apoptosis and cell migration were no significance in two bladder cancer cell lines, T24 and 5637, and human foreskin fibroblast (HFF) cells. The aRNA significantly inhibited the expression of *MYC* in mRNA and protein levels, as well as the proliferation and migration of the cancer cells, but not in HFF cells. These results indicated that regulatory RNAs selectively controlled the expression of amiRNAs and ultimately suppress the progression of bladder cancer cells without affecting normal cells. Synthetic regulatory RNAs might be a selective therapeutic approach for bladder cancer.

## Introduction

Bladder cancer is one of the most common tumors all over the world [1]. Surgery, radiation therapy and chemotherapy are major therapies for the treatment of bladder cancer while they have inevitable side effects because of lack of specificity [2, 3]. Thus, we should focus on an effective and tumor-specific therapeutic method for bladder cancer.

MicroRNA (miRNA) is a type of endogenous non-coding RNAs and also a key mediator of RNA interference (RNAi) in eukaryotes [4–6]. Many protein-coding genes are influenced by miRNAs through target-sequence interaction [4]. Complete or partial pairing of the miRNA complex to the target mRNA transcript causes RNA degradation or translational inhibition [7]. Studies show that based on the pri-miR-155 backbone, artificial miRNAs (amiRNAs) were created through inserting a series of miRNA precursors with different stem sequences, which generated efficient mature miRNA [8, 9]. In addition, a subset of non-coding RNAs, regulatory RNAs, repress or activate translation through sensing environmental signals or other RNA molecules [10].

GAL4, a yeast transcriptional activator, and upstream activating sequence (UAS) that is the specific recognition sequence of GAL4 or GAL4-VP64 fusion protein, are two components in GAL4/UAS system [11]. This system operates not only in yeast but also in various mammal cells [12, 13]. The UAS, a minimal promoter, cannot drive the expression of downstream targeting genes until the binding of GAL4 protein or GAL4-VP64 fusion protein [13]. Moreover, to develop a kind of cancer-specific treatment, we try to use tumor-specific elements to achieve this purpose. The human telomerase reverse transcriptase (hTERT), one of the subunits of telomerase, exists in most tumors but not normal tissues [14, 15]. Previous studies show that compared with the wild-type *hTERT* promoter, the mutant *hTERT* promoter could enhance the expression of *hTERT* or downstream genes and still maintain its tumor-specific feature [16–18]. We pick the tumor-specific element, mutant *hTERT* promoter, from a previous study [19]. In their study, they utilized the mutant *hTERT* to drive expression of BCL2 shRNA. However, their driven efficiency was still not very high and stable expression of BCL2 shRNA may do harm to cells. Compared with their work, we may overcome these limitations.

Using the above elements, we could construct artificial devices with novel functions according to the principles of synthetic biology [17, 20, 21]. Synthetic devices have been used to regulate gene expression or control the biological phenotypes of cancer cells [22, 23]. Synthetic amiRNA, one of the synthetic devices, could knockdown expression of genes with several advantages, including co-expression with a gene of interest, stable expression and low toxicity [24–28]. Wang, et al. synthesized amiRNA clusters and used them as powerful tools for multiplex gene knockdown at the posttranscriptional level [9]. In this study, we construct and synthesize regulatory RNAs to control the gene expression. The inhibitive RNA (iRNA) binds UAS so GAL4-VP64 cannot recognize UAS. The active RNA (aRNA) which is constructed according to the previous study [10] interacts with iRNA tightly and UAS is exposed again, and finally GAL4-VP64 binds UAS to activate amiRNAs targeting MYC. *MYC* was one of the most well-known deregulated oncogenes and the third most amplified gene in human cancer [29, 30]. In bladder cancer, increase of *MYC* copy number occurred before muscle invasion and correlated with grade [31]. Furthermore, MYC was regarded as an independent predictor of progression-free and cancer-specific survival [32]. Thus, we choose MYC as the therapeutic target in this study.

In our study, we constructed synthetic artificial miRNA devices driven by UAS to suppress the expression of the *MYC* oncogene in bladder cancer. As mentioned above, synthetic iRNA block UAS from binding the GAL4-VP64 fusion protein. And results of in vitro and in vivo experiments showed that the GAL4-VP64 fusion protein interacts with UAS again when aRNA expressed. In short, synthetic regulatory RNAs selectively inhibit the progression of bladder cancer through controlling the expression of amiRNAs targeting MYC.

## Materials and methods

### Cell lines and cell culture

Human bladder cancer cell lines (T24 and 5637) and human foreskin fibroblast (HFF) cells were purchased from the Institute of Cell Research, Chinese Academic of Sciences, Shanghai, China. The normal bladder epithelium SV-HUC-1 cell line was established by transformation of human normal ureter tissue with SV40 virus, and purchased from American Type Culture Collection (ATCC). T24 and HFF cells were cultured in DMEM (Invitrogen, Carlsbad, CA, USA) with 10% fetal bovine serum (FBS). The 5637 cells were maintained in 10% FBS RPMI-1640 media (Invitrogen, Carlsbad, CA, USA). The SV-HUC-1 cells were grown according to the manufacturer’s protocol. The cells were cultured at 37 °C in a humidified atmosphere of 5% CO_2_ in an incubator.

### Patient samples

Thirty-nine pairs of bladder cancer tissues and matched para-carcinoma tissues were resected from patients diagnosed with bladder cancer. Samples were treated with other necessary procedures according to a previous study [33]. This study was admitted by the Institutional Review Board of Peking University Shenzhen Hospital.

### Creation of iRNA, aRNA and artificial miRNAs

To construct a vector that expresses iRNA, the sequence of iRNA was inserted into the pcDNA3-EGFP vector (Addgene #13031) between the restriction sites XhoI and XbaI. To create vectors expressing aRNA, we used the mutant *hTERT* promoter and aRNA to replace CMV promoter and EGFP respectively in the pcDNA3-EGFP vector. GAL4-VP64 displaced EGFP in pcDNA3-EGFP vector to create pcDNA3-GAL4-VP64 vector. Besides, UAS and related artificial microRNAs were designed, synthesized and inserted between the restriction sites BbsI and BstBI into the pcDNA3-GAL4-VP64 vector. In dual luciferase reporter assays, UAS replaced the SV40 promoter in the siCHECK™-2 vector (Promega, Madison, USA) between the restriction sites BgIII and Nhel. The iRNA can bind UAS while aRNA driven by mutant *hTERT* promoter can interact with iRNA. The siRNA duplexes for *MYC* and the negative control (indicated as si-*MYC* and si-NC) were designed and synthesized by GenePharma, Suzhou, China. All of the related sequences were shown in Table 1.

**Table 1:** Relative sequences in this study

Name	Relative sequence (5′-3′)
NC amiRNA	CTCGAGAAGGTATATTGCTGTTGACAGTGAGCGCATTCTCCGAACGTGTCACGTATAGTG AAGCCACAGATGTATACGTGACACGTTCGGAGAATTTGCCTACTGCCTCGCTTCAAGGTA TATTGCTGTTGACAGTGAGCGCATTCTCCGAACGTGTCACGTATAGTGAAGCCACAGATG TATACGTGACACGTTCGGAGAATTTGCCTACTGCCTCGGCGGCCGC
*MYC* amiRNA	CTCGAGAAGGTATATTGCTGTTGACAGTGAGCGCACAGAAATGTCCTGAGCAATATAGTG AAGCCACAGATGTATATTGCTCAGGACATTTCTGTTTGCCTACTGCCTCGCTTCCTTCCTTC AAGGTATATTGCTGTTGACAGTGAGCGCATGGACAGTGTCAGAGTCTATAGTGAAGCCAC AGATGTATAGACTCTGACACTGTCCATTTGCCTACTGCCTCGGCGGCCGC
iRNA	GAAUUCCGGAGGUCAGAACUCUUGG
aRNA	CGCCAAGAGUUCUGUCCUCCGGUGGUGGUUAAUGAAAAUUAACUUACUAUACCAUAUA UCUCUAGA
mutant hTERT promoter	GGCCCCTCCCTCGGGTTACCCCACAGCCTAGGCCGATTCGACCTCTCTCCGCTGGGGCC CTCGCTGGCGTCCCTGCACCCTGGGAGCGCGAGCGGCGCGCGGGCGGGGAAGCGCGG CCCAGACCCCCGGGTCCGCCCGGAGCAGCTGCGCTGTCGGGGCCAGGCCGGGCTCCCA GTGGATTCGCGGGCACAGACGCCCAGGACCGCGCTCCCCACGTGGCGGAGGGACTGGG GACCCGGGCACCCGTCCTGCCCCTTCACCTTCCGGCTCCGCCTCCTCCGCGCGGACCCCG CCCCGTCCCGACCCCTTCCGGGTTTCCGGCCCAGCCCCTTCCGGGCCCTCCCAGCCCCTC CCCTTCCTTTCCGGGGCCCCGCCCTCTCCTCGCGGCGCGAGTTTCCGGCAGCGCTGCGTC CTGCTGCGCACGTGGGAAGCCCTGGCCC CGGCCACCCCCGCG
UAS	CGGAGTACTGTCCTCCG
GAL4-VP64	ATGAAGCTACTGTCTTCTATCGAACAAGCATGCGATATTTGCCGACTTAAAAAGCTCAAG TGCT CCAAAGAAAAACCGAAGTGCGCCAAGTGTCTGAAGAACAACTGGGAGTGTCGCT ACTCTCCCAAAACCAAAAGGTCTCCGCTGACTAGGGCACATCTGACAGAAGTGGAATCA AGGCTAGAAAGACTGGAACAGCTATTTCTACTGATTTTTCCTCGAGAAGACCTTGACATG ATTTTGAAAATGGATTCTTTACAGGATATAAAAGCATTGTTAACAGGATTATTTGTACAAGA TAATGTGAATAAAGATGCCGTCACAGATAGATTGGCTTCAGTGGAGACTGATATGCCTCTA ACATTGAGACAGCATAGAATAAGTGCGACATCATCATCGGAAGAGAGTAGTAACAAAGGT CAAAGACAGTTGACTGTATCGGGTTCCGGACGGGCTGACGCATTGGACGATTTTGATCTG GATATGCTGGGAAGTGACGCCCTCGATGATTTTGACCTTGACATGCTTGGTTCGGATGCCC TTGATGACTTTGACCTCGACATGCTCGGCAGTGACGCCCTTGATGATTTCGACCTGGACAT GCTGATTAAC
si-*MYC* sense si-*MYC* antisense	GCUUCACCAACAGGAACUATT UAGUUCCUGUUGGUGAAGCTT

### Cell transfection

The propagated synthetic constructed vectors from E.coli bacteria were extracted using Plasmid Midiprep kits (Promega, Madison, USA). The cells were transfected with specific siRNA or synthetic vectors using Lipofectamine 2000 Transfection Reagent (Invitrogen, Carlsbad, CA, USA) according to the manufacturer’s instructions.

### Dual luciferase reporter assay

Cells (1 × 10^5^ per well) were cultured in 24-well plates and transfected with specific siRNA or vectors. After 48 h transfection, the luciferase activity was measured using the dual luciferase assay system (Promega, Madison, WI, USA) according to the manufacturer’s protocol. The experiments were performed at least three times.

### RNA extraction and quantitative RT-PCR

The TRIzol reagent (Invitrogen, Grand Island, NY, USA) was used to extract total RNA from cells after transfection according to the manufacturer’s protocol. The cDNA was synthesized from total RNA using PrimeScript RT Reagent Kit with gDNA Eraser (Takara, Dalian, China). The mRNA expression levels of *MYC* were measured by quantitative RT-PCR (qRT-PCR) on the Roche lightcycler 480 Real-Time PCR System. GAPDH was used as the endogenous control to normalize the data. The primers used were: *MYC*-forward: 5′-GCAGCTGCTTAGACG CTGGA-3′, *MYC*-reverse: 5′-CGCAGTAGAAATACGGCTGCAC-3′; GAPDH –forward: 5′-CGCTCTCTGCTCCTCCTGTTC-3′, GAPDH-reverse: 5′-ATCCGTT GACTCCGACCTTCAC-3′. The comparative ΔCt method was used to analyze the relative expression of *MYC*. All of the experiments were performed at least three times.

### Western-blot analysis

The transfected cells were washed in PBS and lysed in RIPA reagent (Beyotime, Jiansu, China). The bicinchoninic acid quantification assay (Pierce Biotechnolofy, Rockford, IL, USA) was used to calculate the protein concentration. Equal amounts of whole protein extract were electrophoresed on SDS-polyacrylamide gels and transferred to polyvinylidene difluoride membranes using a semi-dry transfer cell (Bio-Rad Laboratories, Hercules, CA, USA). After blocked with 5% milk, the membranes were incubated over night with specific primary antibodies against MYC (1:1000; Cell Signaling Technology, USA) and GAPDH (1:10,000; Sigma-Aldrich). Horseradish peroxidase-conjugated secondary antibody (Amersham, Piscataway, NJ, USA) was used to incubate the blot for one hour at room temperature on a rocking platform. Finally, signal intensities were quantified using Super Signal chemiluminescence reagents (Pierce).

### Cell proliferation assay

Cell Counting Kit-8 (Beyotime Institute of Biotechnology, Shanghai, China) was used to detect cell proliferation according to the manufacturer’s instructions. Cells (4000 per well) were seeded into a 96-well plate. Then, 24, 48 or 72 h after transfection, 10 μl of CCK-8 was added to each well and the cells were incubated for 40 min. Absorbance was detected at a wavelength of 450 nm using an ELISA microplate reader (Bio-Rad, Hercules, CA, USA). All of the assays were performed in triplicate.

### ELISA assay

Cells were transfected with specific siRNA or vectors. The activity of caspase-3 represented the levels of apoptosis and was measured using the caspase 3 enzyme-linked immunosorbent assay (ELISA) assay kit (Hcusabio, Wuhan, China) according to the manufacturer’s protocols. All of the experiments were performed at least three times.

### Cell migration assay

Cells were seeded in 6-well plates to 90% confluence before transfection. A sterile pipette tip was used to create a clear line. Twenty-four hours after transfection, the migration distance was measured using the software program HMIAS-2000. The experiments were repeated at least three times.

### Xenograft model of tumor growth in vivo

The experimental procedures were approved by Institutional Ethics Review Board. Male immune-deficient BALB/c nude mice (4–5 weeks old) were purchased from Beijing Wei-tong Li-hua Laboratory Animals and Technology Ltd., Beijing, China. Vectors were packed into lentivirus according to the manufacturer’s protocols using Lentiviral Packing Kit, SyngenTech, China. In detail, 10^7^ 5637 cells were suspended in 100 μl Matrigel (BD Biosciences, Franklin Lakes, NJ, USA) and injected subcutaneously into the right armpits of BALB/c nude mice. LV-NC represents NC amiRNA + GAL4-VP64 + iRNA + aRNA. Besides, *MYC* amiRNA + GAL4-VP64+ iRNA + aRNA was regarded as the LV-Treatment group. 15 days after implantation, tumor volumes were monitored every 5 days over a 2-week period. Tumor volumes were calculated using the formula: 0.5 × length × width^2^. At the end of the experiment, mice were euthanized, and the subcutaneous weight of each tumor was measured.

### Statistical analysis

All statistical analyses were performed using 21.0 version SPSS computer software (SPSS Inc., Chicago, IL, USA). The data are presented as the mean ± S.D. and were analyzed using Student’s *t*-test or ANOVA. A two-sided value of *P* < 0.05 was considered statistically significant.

## Results

### *MYC* is an oncogene in bladder cancer

**Fig. 1:**
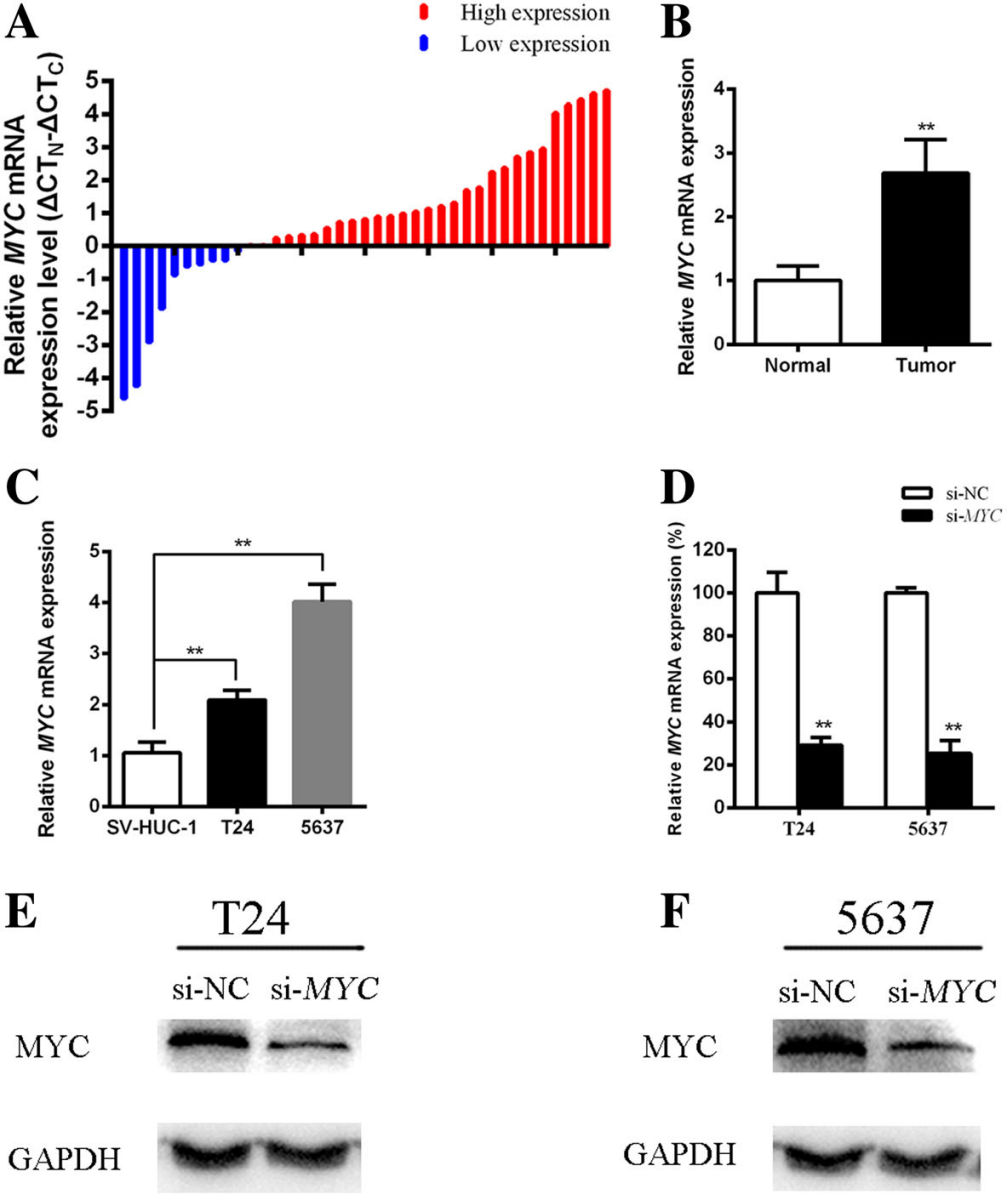
The expression of MYC was upregulated in bladder cancer tissues and cells, and siRNA was able to knockdown the expression of this gene in cells. (**a**) qRT-PCR was used to detect the expression of *MYC* in bladder cancer tissues. ΔCT_N_ means comparative CT in normal tissue. ΔCT_C_ represents comparative CT in tumor tissue. (**b, c**) Compared with the negative control, the expression of *MYC* mRNA was significantly increased in bladder cancer tissues (**b**, *P* < 0.01), bladder cancer cell lines T24 and 5637 (C, P < 0.01). (**d**) The mRNA expression level of *MYC* was statistically decreased after transfection of si-*MYC* in T24 and 5637 cells (both P < 0.01). (**e, f**) The protein expression level of MYC was statistically decreased after transfection of si-*MYC* in T24 (**e**) and 5637 (**f**) (both P < 0.01). All data are shown as the mean ± SD (*p < 0.05, **p < 0.01); bar, SD

**Fig. 2:**
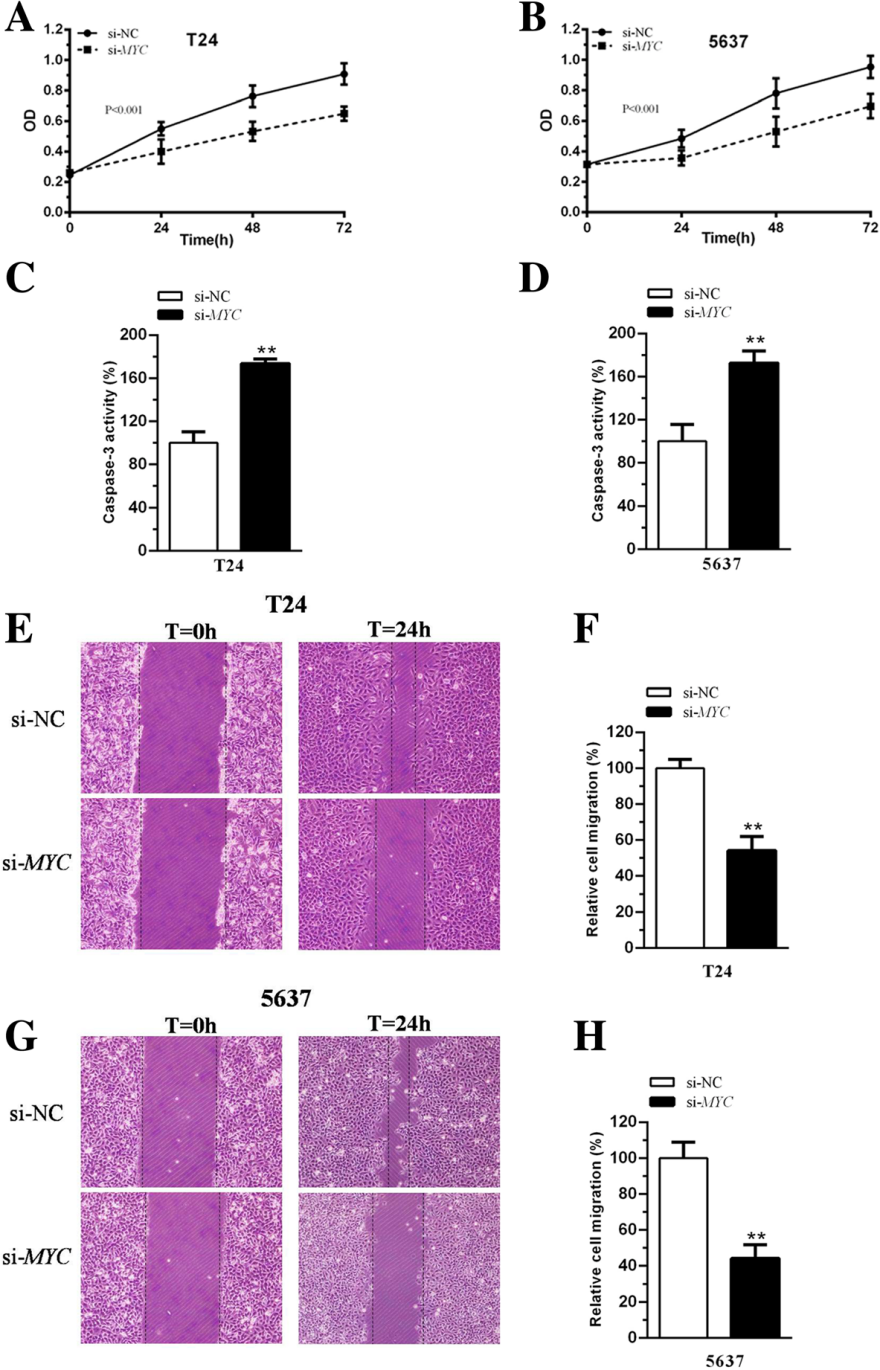
Effects of MYC on cell proliferation, apoptosis and cell migration in bladder cancer cells. (**a, b**) Compared with the si-NC group, cell growth was inhibited significantly in the si-*MYC* group in T24 (A) and 5637 (B) (both *P* < 0.01). (**c, d**) Compared with the si-NC group, cell apoptosis was statistically increased in the si-*MYC* group in T24 (**c**) and 5637 (D) (both P < 0.01). (**e, f**) Compared with si-NC, cell migration was significantly suppressed after transfection of si-*MYC* in T24. (**g, h**) Compared with the negative control, cell motility was significantly inhibited after transfection of si-*MYC* in 5637. All data are shown as the mean ± SD (*p < 0.05, **p < 0.01); bar, SD

Compared with normal counterparts, the *MYC* mRNA expression level was increased significantly in 74.4% (29 of 39) of tumor tissues (Fig. 1A). The expression of *MYC* in tumors was 2.8 times higher than in para-cancer tissues (Fig. 1B, P < 0.01). Compared with the SV-HUC-1 cell line, *MYC* expression was upregulated significantly in T24 and 5637 (Fig. 1C, both *P* < 0.01). As shown in Table 2, high expression of *MYC* was significantly correlated with bladder cancer histological grade (*P* = 0.031) and TNM stage (*P* = 0.016). However, there was no association between age, gender, tumor size, lymph node metastasis and *MYC* expression level. To investigate the function of this gene in bladder cancer cells, we synthesized specific siRNA duplexes to knockdown *MYC* expression. The qRT-PCR results showed that si-*MYC*, but not si-NC, can dramatically decrease the expression of *MYC* in T24 and 5637 (Fig. 1D, both *P* < 0.01). The Western-blot analysis assay demonstrated that compared with the protein level of MYC in si-NC group, the protein level was significantly suppressed in si-*MYC* group in T24 (Fig. 1E) and 5637 (Fig. 1F). After inhibition of *MYC* expression, cell growth was arrested significantly in T24 and 5637 (Fig. 2A and B, both *P* < 0.001). Additionally, cell apoptosis was significantly promoted after suppression of *MYC* expression in T24 and 5637 (Fig. 2C and D, both *P* < 0.01). In addition, cell migration was repressed after transfection with si-*MYC* (Fig. 2E, F, G and H, both P < 0.01). These data suggest that *MYC* should be an oncogenic factor in bladder cancer cells.

**Table 2:** Correlation between *MYC* expression and clinicopathological characteristics of bladder cancer patients

Parameters	Group	Total	*MYC* expression		*P* value
			High	Low	
Age (years)	<59	12	7	5	0.232
	≥59	27	22	5	
Gender	Male	25	18	7	0.721
	Female	14	11	3	
Tumor size (cm)	<3	21	15	6	0.726
	≥3	18	14	4	
Histological grade (PUNLMP)	Low-grade	19	11	8	0.031*
	High-grade	20	18	2	
TNM stage	0/ I	8	3	5	0.016*
	II/III/IV	31	26	5	
Lymph nodes metastasis	N0	33	25	8	0.636
	N1 or above	6	4	2	

*PUNLMP* papillary urothelial neoplasm of low malignant potential, *TNM* according to the seventh edition of staging TNM of Union Internationale Contre Le Cancer (UICC) in 2009

*P < 0.05 was considered significant (Chi-square test between 2 groups)

### Schematic strategy for this study and luciferase assay that was used to validate the roles of aRNA and iRNA in bladder cancer cells

**Fig. 3:**
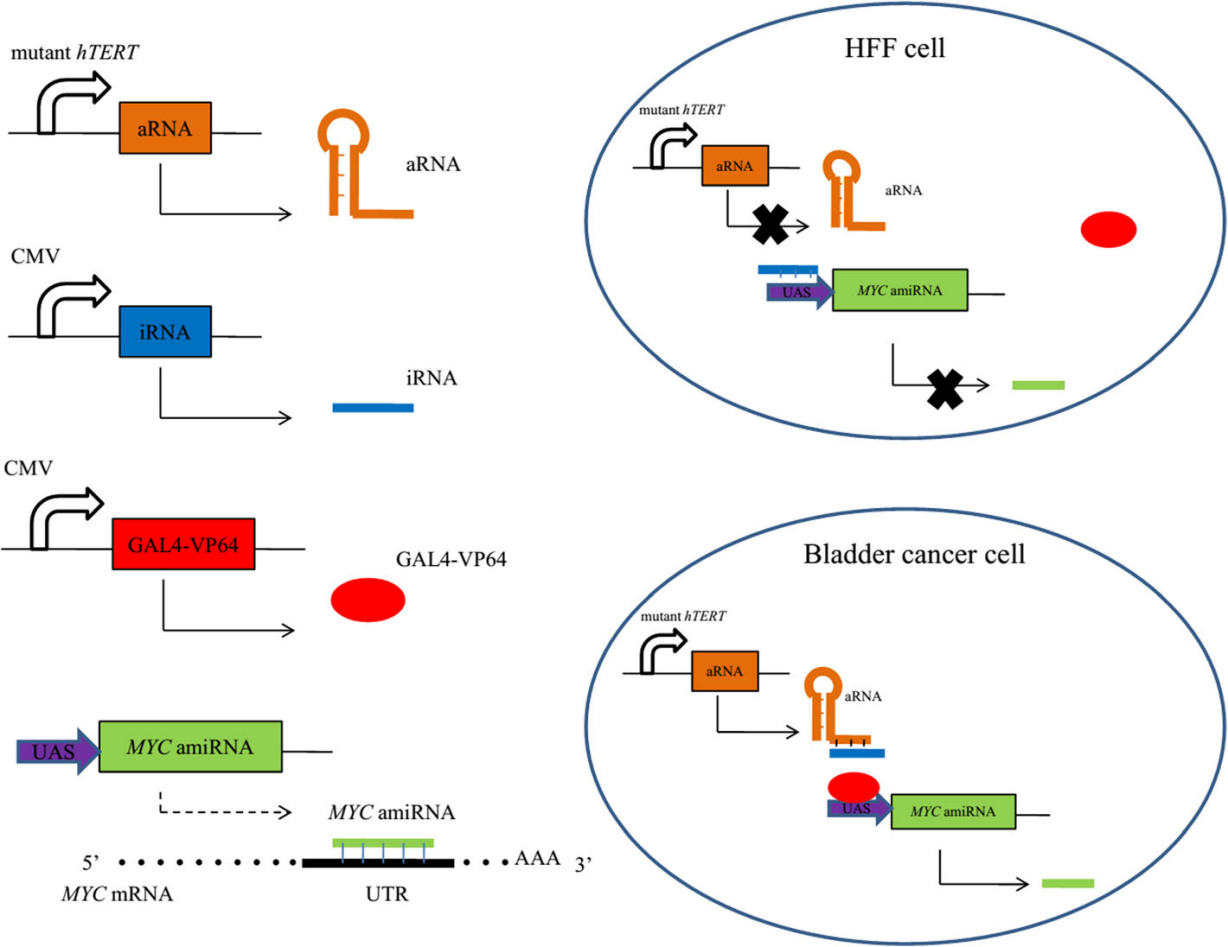
A schematic of the mechanism of synthetic regulatory RNAs. The mutant *hTERT* promoter drives the expression of aRNA. The amiRNA targeting *MYC* is driven by UAS. In HFF cells, aRNA cannot be expressed. The iRNA interacts with UAS so GAL4-VP64 cannot bind UAS to activate the expression of amiRNA. Artificial miRNAs can generate miRNAs that interact with the 3′ UTR of *MYC* mRNA completely and causes *MYC* mRNA degradation. However, aRNA is overexpressed in bladder cancer cells. The aRNA can interact with iRNA so UAS is exposed to GAL4-VP64 again. Thus, *MYC* amiRNA will be driven by the UAS and GAL4-VP64 complex

**Fig. 4:**
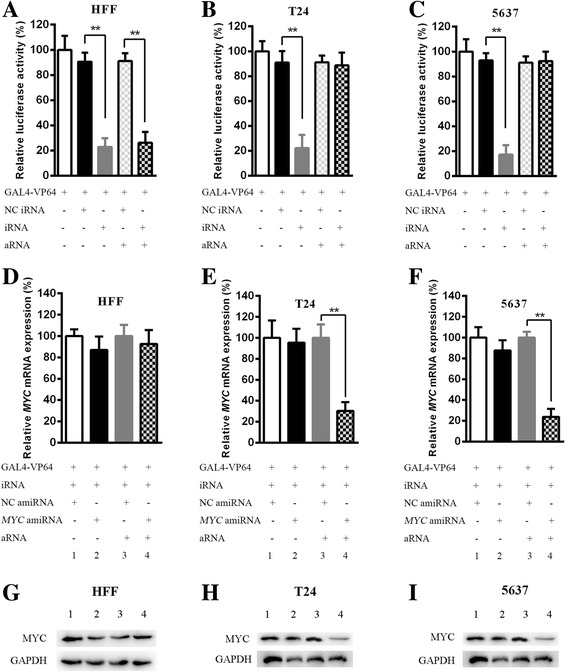
Effects of synthetic regulatory RNAs on luciferase activity, and *MYC* mRNA and protein levels. (**a, b** and **c**) When aRNA was not expressed in the three cell lines, the luciferase activity was significantly decreased between the NC iRNA group and iRNA group in T24, 5637 and HFF. However, the luciferase activity was still significantly decreased in HFF (**a**) after addition of aRNA in cells between these two groups. But the luciferase activity was no statistical difference in T24 (**b**) and 5637 (**c**). (**d, e, f, g, h** and **i**) Without aRNA, there was no difference between column 1 and 2 in all three cell lines and therefore the transcriptional and translational levels of *MYC* was not inhibited. After the cells were transfected with aRNA as shown in T24 and 5637, the mRNA and protein levels of *MYC* was largely reduced by expression of *MYC* amiRNA (column 4 in E, F, H and I) compared with the negative group (column 3 in **e, f, h** and **i**). However, there was no significant difference between column 3 and 4 (**d** and **g**) in HFF. All data are presented as the mean ± SD (*p < 0.05, **p < 0.01); bar, SD

The schematic strategy for the expression of the synthetic artificial miRNA to target oncogenic *MYC* was shown in Fig. 3. The amiRNA cannot be expressed without aRNA when the cells were only transfected with GAL4-VP64 and iRNA. The aRNA driven by the tumor-specific mutant *hTERT* promoter could be expressed as expected only in bladder cancer cells but not in HFF cells. Artificial miRNAs can generate miRNAs that bind the 3′ UTR of *MYC* mRNA completely and causes *MYC* mRNA degradation. Thus, we can use this strategy to control the expression of *MYC* in bladder cancer cells. To verify whether iRNA can bind aRNA and UAS, we transfected relevant vectors (UAS-luciferase, GAL4-VP64, iRNA and aRNA) into cells and performed luciferase assays. When aRNA was not expressed in these three cell lines, the luciferase activity was significantly decreased between the NC iRNA group and iRNA group in T24, 5637 and HFF (Fig. 4A, B and C, P < 0.05). The results showed that iRNA can bind UAS so that GAL4-VP64 cannot interact with UAS to activate the expression of luciferase. After transfection of aRNA in cells, the luciferase activity did not change in T24 and 5637 between NC iRNA group and iRNA group (Fig. 4B and C, P > 0.05). However, it was still dramatically decreased in HFF (Fig. 4A, P < 0.01). These data suggested that aRNA can bind iRNA to expose UAS again and reverse the repressive effects of iRNA on luciferase activity in T24 and 5637, but not HFF.

### The mRNA levels and protein levels of *MYC* were selectively inhibited by synthetic regulatory RNAs in bladder cancer cells

As shown in subsequent validated experiments (Fig. 4D, E and F), in the absence of aRNA, amiRNAs (NC amiRNA and *MYC* amiRNA) were not expressed in all three cell lines and therefore could not inhibit the transcriptional level and protein level of MYC (column 1 and 2 in Fig. 4D, E, F, G, H and I). After cells were transfected with aRNA driven by the mutant *hTERT* promoter, the aRNA was only expressed in T24 and 5637, thus interacting with iRNA to expose UAS to allow GAL4-VP64 to combine with UAS. Expectedly, as shown in T24 and 5637, the mRNA level of *MYC* was largely reduced by expressed *MYC* amiRNA (column 4 in Fig. 4E, F) compared with the negative group (column 3 in Fig. 4E and F). However, there was no significant difference between column 3 and 4 in Fig. 4D (*P* > 0.05). Besides, similar results were shown in the protein levels of MYC. There was no obvious difference between column 3 and 4 in HFF (Fig. 4G). However, the protein levels of MYC were significantly inhibited in column 4 group compare with column 3 in T24 (Fig. 4H) and 5637 (Fig. 4I). These results demonstrated that this synthetic system can selectively control the expression of the *MYC* oncogene in bladder cancer T24 and 5637 cell lines without affecting HFF.

### Selective inhibition of cell growth by synthetic regulatory RNAs in bladder cancer cells

**Fig. 5:**
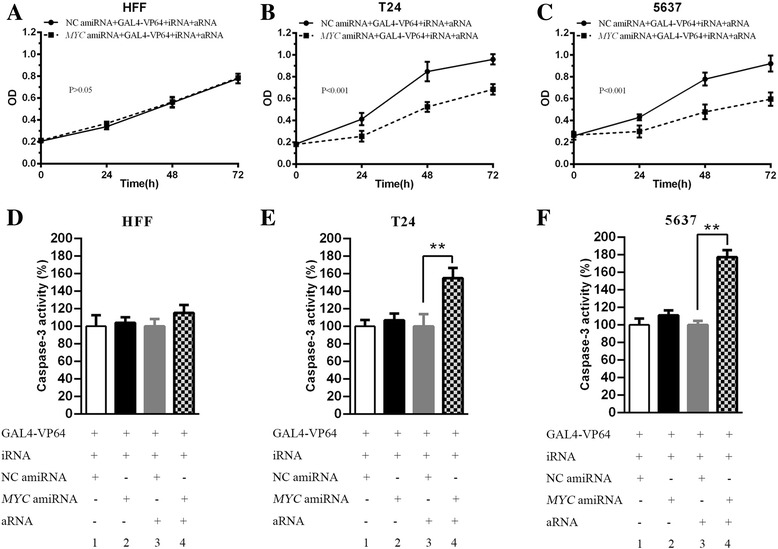
Effects of synthetic regulatory RNAs on cell proliferation and apoptosis. (**a, b** and **c**) Cells were seeded in 96-well plates and transfected with various vectors that were packed into lentivirus. ANOVA was used to analyze the data. There was no difference between the NC amiRNA + GAL4-VP64 + iRNA + aRNA group and the *MYC* amiRNA + GAL4-VP64 + iRNA + aRNA group in HFF (**a**). Cell proliferation was significantly decreased in T24 (**b**) and 5637 (**c**) between these two groups. (**d, e** and **f**) Without aRNA, there was no significant difference in the caspase-3 activity between column 1 and 2 in HFF (D), T24 (**e**) and 5637 (**f**). When the cells were transfected with aRNA driven by the mutant *hTERT* promoter, the caspase-3 activity was statistically increased in column 4 compared with column 3 in T24 (**e**) and 5637 (**f**) (both *P* < 0.01). However, no difference was present in HFF between column 3 and 4 (D). All data are shown as the mean ± SD (*p < 0.05, **p < 0.01); bar, SD

A CCK-8 assay was performed to detect cell proliferation. T24, 5637, and HFF were transfected with vectors that were packed into lentivirus in 96-well plates. The CCK-8 assay showed that there was no significant difference between the NC amiRNA + GAL4-VP64 + iRNA + aRNA group and the *MYC* amiRNA + GAL4-VP64 + iRNA + aRNA group in HFF (Fig. 5A, P > 0.05). However, cell proliferation was suppressed significantly between these two groups in T24 and 5637 (Fig. 5B and C, P < 0.001). The results indicated that cell growth was selectively inhibited by synthetic regulatory RNAs in bladder cancer cells.

### Selectively inducing apoptosis by synthetic regulatory RNAs in bladder cancer cells

To investigate the effect of artificial miRNA and regulatory RNA on the apoptosis of bladder cancer cells and HFF cells, an ELISA assay was performed to measure the apoptosis rate. Without the expression of aRNA, the caspase-3 activity was not different between group 1 (column 1) and group 2 (column 2) in HFF, T24 and 5637 (Fig. 5D, E and F, P > 0.05). When aRNA driven by a mutant *hTERT* promoter was added, the caspase-3 activity did not significantly change between group 3 (column 3) and group 4 (column 4) in HFF. However, the caspase-3 activity increased statistically between group 3 and 4 in T24 and 5637 (Fig. 5E and F, P < 0.01). The data showed that cell apoptosis was selectively promoted by synthetic regulatory RNAs in bladder cancer cells.

### Selective inhibition of cell migration by synthetic regulatory RNAs in bladder cancer cells

**Fig. 6:**
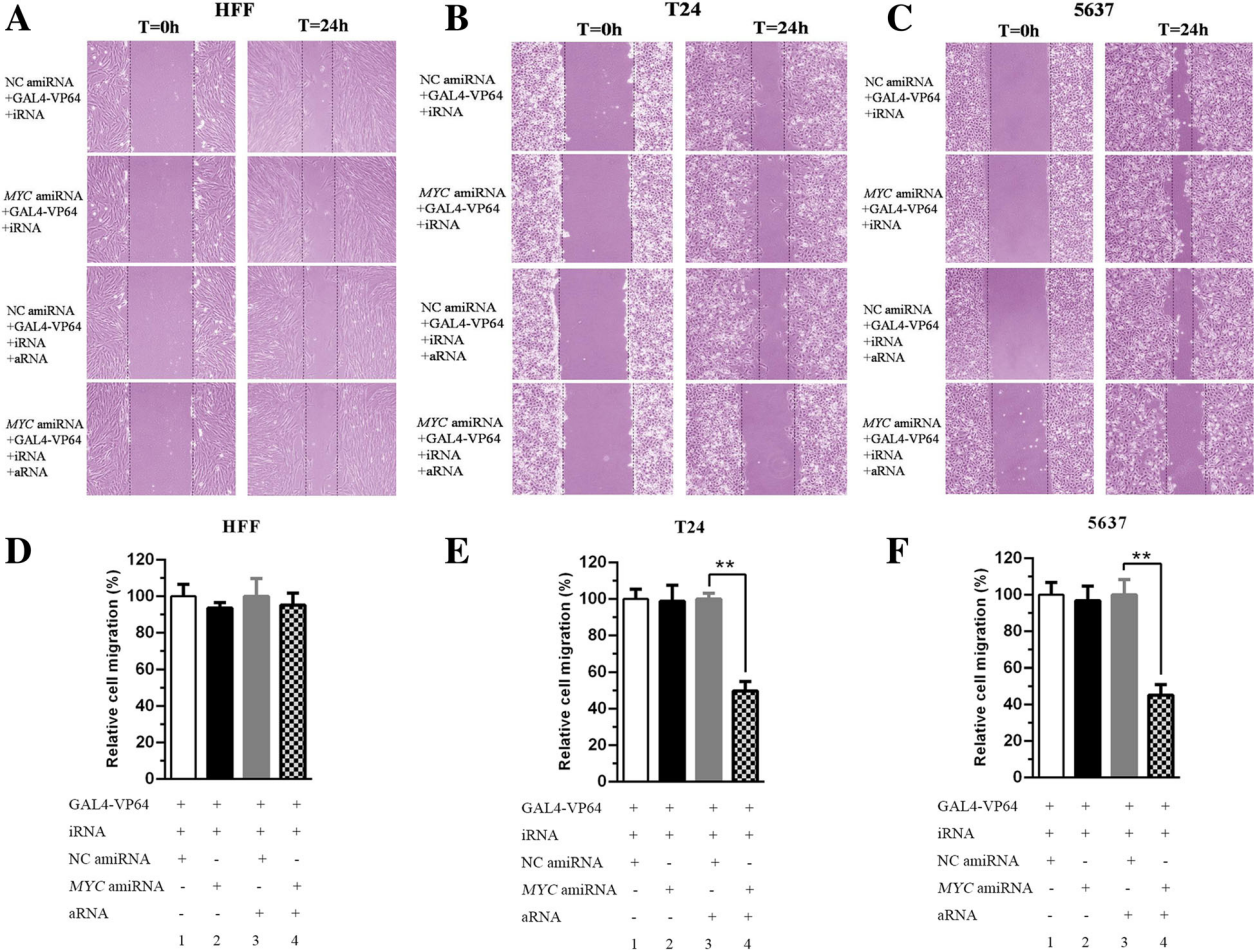
Effects of synthetic regulatory RNAs on cell migration. Representative images of each group were shown in HFF (**a**), T24 (**b**) and 5637 (**c**). Without the expression of aRNA, compared with the NC amiRNA + GAL4-VP64 + iRNA group, the relative cell migration was no significant difference in the *MYC* amiRNA + GAL4-VP64 + iRNA group in HFF, T24 and 5637 (**a, b , c, d, e** and **F**, *P* > 0.05). After expression of aRNA, there was no statistical change in cell migration between these two groups in HFF (**a** and **d**, P > 0.05). However, cell migration was significantly decreased in the *MYC* amiRNA + GAL4-VP64 + iRNA + aRNA group (column 4) compared with the NC amiRNA + GAL4-VP64 + iRNA + aRNA group (column 3) in T24 and 5637 (**b, c, d** and **f**, P < 0.01). All data are shown as the mean ± SD (*p < 0.05, **p < 0.01); bar, SD

A cell scratch assay was used to measure cell motility in 6-well plates according to the manufacturer’s protocol. Without the expression of aRNA, compared with the NC amiRNA + GAL4-VP64 + iRNA group, the relative cell migration was not significantly different in the *MYC* amiRNA + GAL4-VP64 + iRNA group in HFF, T24 and 5637 (Fig. 6A, B, C, D, E and F, P > 0.05). When aRNA was added to this system, there was no statistical change between the NC amiRNA + GAL4-VP64 + iRNA + aRNA group and the *MYC* amiRNA + GAL4-VP64 + iRNA + aRNA group in HFF (Fig. 6A and D, P > 0.05). However, cell migration was significantly decreased in the *MYC* amiRNA + GAL4-VP64 + iRNA + aRNA group (column 4) compared with the NC amiRNA + GAL4-VP64 + iRNA + aRNA group (column 3) in T24 and 5637 (Fig. 6B, C, E and F, P < 0.01). The cell migration was inhibited by approximately 50% in T24 (Fig. 6E, P < 0.01) and approximately 55% in 5637 (Fig. 6F, P < 0.01). The cell migration results revealed that cell mobility was selectively suppressed by synthetic regulatory RNAs in bladder cancer cells.

### Knockdown of MYC by synthetic regulatory RNAs inhibited bladder cancer cell growth in vivo

**Fig. 7:**
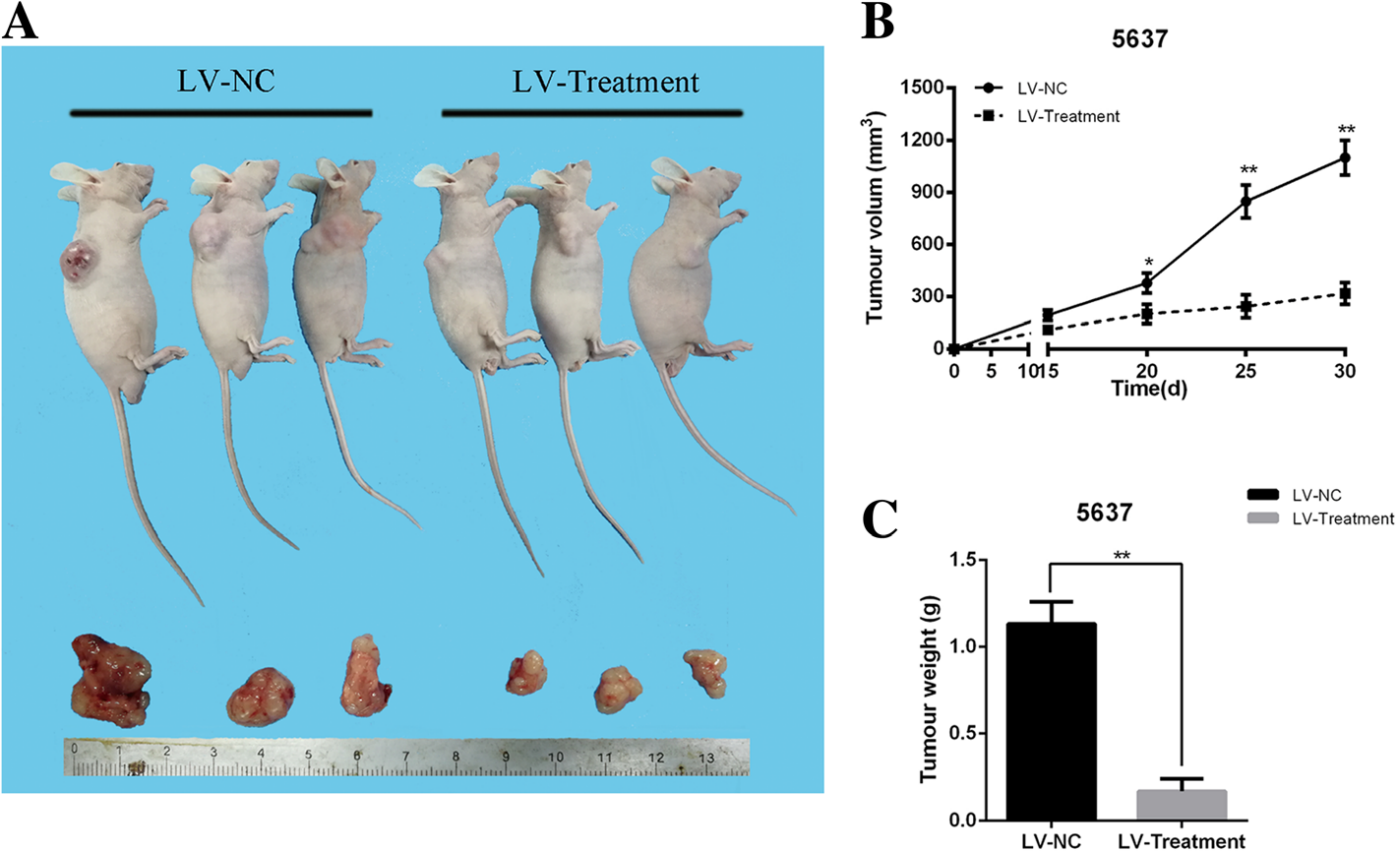
Effects of synthetic regulatory RNAs on cell growth in vivo. (**a**) Representative images of tumor were shown in LV-NC group and LV-Treatment group. LV-NC group represents NC amiRNA + GAL4-VP64 + iRNA + aRNA. LV-Treatment group is on behalf of *MYC* amiRNA + GAL4-VP64+ iRNA + aRNA. (**b**) Compared with the LV-NC group, the tumor volume curve was significantly suppressed in LV-Treatment group. (**c**) Compared with the LV-NC group, the tumor weight in LV-Treatment group was obviously lower than that in LV-NC group (P < 0.01). All data are shown as the mean ± SD (*p < 0.05, **p < 0.01); bar, SD

To examine the knockdown effect of MYC by synthetic regulatory RNAs, vectors were packed into lentvirus. To measure the functional role of synthetic regulatory RNAs in tumor growth in vivo, 5637 cells expressing LV-Treatment or LV-NC were subcutaneously inoculated into nude mice. Thirty days later, tumors were harvested and the tumor volume was significantly smaller than that in LV-Treatment compared with the LV-NC group (Fig. 7A). As shown in Fig. 7B, the tumor volume curve was significantly suppressed in LV-Treatment group. Besides, the tumor weight in LV-Treatment group was obviously lower than that in LV-NC group (Fig. 7C, P < 0.01). In summary, knockdown of MYC by synthetic regulatory RNAs can inhibit bladder cancer cell growth in vivo. These results were consistent with the findings of cell growth assay by CCK-8.

## Discussion

RNA molecules are often regarded as messengers of information from genes to the protein and also have regulatory roles in human diseases [34–37]. Repressor RNA and activator RNA are small RNAs that have regulatory roles in controlling post-transcriptional gene expression in prokaryotic cells [10, 38]. Whether these regulatory RNAs can control gene expression in eukaryotic cells is unknown. In our study, we construct regulatory RNAs, iRNA and aRNA according to the principles of creation in a previous study [10], and it is the first time to measure the roles of regulatory RNAs in mammal cells.

UAS cannot drive the downstream gene expression without the binding of activating proteins, such as GAL4 or GAL4-VP64 fusion protein [13, 39]. In the present study, the iRNA was used to compete with GAL4-VP64 protein to bind UAS. However, synthetic aRNA interacted with iRNA and exposed UAS again. Thus, GAL4-VP64 protein can bind UAS again and the downstream gene of UAS could be expressed.

The amiRNAs were inserted into the downstream of UAS in this project. MicroRNAs (miRNAs) are small non-coding RNAs and play significant roles in many biological processes [5, 40–42]. Studies show that artificial miRNAs can promote gene silencing in a similar manner to natural miRNAs and they have the same efficiency and are more stable and less cytotoxic compared with shRNAs or siRNAs [43, 44]. Artificial miRNAs can be designed and synthesized to silence multiple genes or a cluster of amiRNA sequences can be constructed to efficiently suppress one gene [9]. Owing to the vital oncogenic role of MYC in the metabolism of cancer, we choose MYC as the therapeutic target for future study.

The mutant *hTERT* promoter is a potential tumor-specific element and has been used to selectively drive expression of downstream genes in bladder cancer cells [17]. Therefore, trying to solve limitations in the specificity and effectiveness of treatment in bladder cancer, we also take advantage of this element to selectively control gene expression in bladder cancer cells. In this study, the aRNA was driven by the mutant *hTERT* promoter picked from a previous study [19] and expressed in bladder cancer cells but not human foreskin fibroblast cells. In our work, expression of the amiRNA targeting MYC was only activated when GAL4-VP64 fusion protein binds UAS. We used synthetic regulatory RNAs to control this binding procedure (GAL4-VP64 binds UAS). Compared with their work [19], our strategy has two advantages. The p65 or VPR (a chimeric activator that is composed of the VP64, p65 and Rta domains) showed much higher transcriptional activation efficiency than VP64 [45]. Our strategy is modular and we could replace GAL4-VP64 with GAL4-P65 or GAL4-VPR to get much higher driven efficiency for expression of the downstream gene in the future, which is one merit. What’s more, stable expression of amiRNAs targeting MYC may be harmful for cells and we construct synthetic regulatory RNAs that are regarded as a similarly “switch” to control the expression of amiRNA, which is the other advantage.

We verified the function of *MYC* in bladder cancer cells. High expression of *MYC* was significantly correlated with bladder cancer histological grade and TNM stage. Additionally, functional experiments showed that *MYC* is an oncogene in bladder cancer cells. Then, we constructed tandem amiRNA sequences targeting oncogenic *MYC* in bladder cancer driven by UAS and tested whether synthetic regulatory RNAs can regulate the expression of amiRNA. Our results demonstrated that when cells express iRNA and GAL4-VP64 protein in bladder cancer cells and HFF cells, the expression of *MYC* cannot be significantly inhibited. When aRNA driven by a tumor-specific promoter was transiently transfected into cells, amiRNA targeting *MYC* can be expressed to markedly decrease the mRNA and protein expression levels of oncogenic *MYC* and significantly inhibit cell growth in vitro, induce apoptosis and suppress the migration of bladder cancer cells, but not human foreskin fibroblast cells. What’s more, the in vivo experiment showed that expression of aRNA can inhibit tumor volume compared with the relative negative control.

In conclusion, we used a synthetic platform to design and construct synthetic regulatory RNAs and artificial miRNAs. We can selectively control the expression of synthetic artificial miRNAs to inhibit progression of bladder cancer by regulatory iRNA and aRNA in vitro and in vivo. Synthetic regulatory RNAs might be a selective therapeutic method for bladder cancer.
